# Keystone nonconsumptive effects within a diverse predator community

**DOI:** 10.1002/ece3.3392

**Published:** 2017-10-28

**Authors:** Amanda J. Meadows, Jeb P. Owen, William E. Snyder

**Affiliations:** ^1^ Department of Entomology Washington State University Pullman WA USA

**Keywords:** aquatic predator, biodiversity, complementarity, *Culex pipiens*, field experiment, trait‐mediated effect

## Abstract

The number of prey killed by diverse predator communities is determined by complementarity and interference among predators, and by traits of particular predator species. However, it is less clear how predators' nonconsumptive effects (NCEs) scale with increasing predator biodiversity. We examined NCEs exerted on *Culex* mosquitoes by a diverse community of aquatic predators. In the field, mosquito larvae co‐occurred with differing densities and species compositions of mesopredator insects; top predator dragonfly naiads were present in roughly half of surveyed water bodies. We reproduced these predator community features in artificial ponds, exposing mosquito larvae to predator cues and measuring resulting effects on mosquito traits throughout development. Nonconsumptive effects of various combinations of mesopredator species reduced the survival of mosquito larvae to pupation, and reduced the size and longevity of adult mosquitoes that later emerged from the water. Intriguingly, adding single dragonfly naiads to ponds restored survivorship of larval mosquitoes to levels seen in the absence of predators, and further decreased adult mosquito longevity compared with mosquitoes emerging from mesopredator treatments. Behavioral observations revealed that mosquito larvae regularly deployed “diving” escape behavior in the presence of the mesopredators, but not when a dragonfly naiad was also present. This suggests that dragonflies may have relaxed NCEs of the mesopredators by causing mosquitoes to abandon energetically costly diving. Our study demonstrates that adding one individual of a functionally unique species can substantially alter community‐wide NCEs of predators on prey. For pathogen vectors like mosquitoes, this could in turn influence disease dynamics.

## 1 INTRODUCTION

Ecologists have long been interested in how the impacts of multiple predator species sum to affect the net number of prey that are killed (e.g., Hairston & Hairston, 1997; Schmitz, 2007; Sih, Englund, & Wooster, 1998). This work has revealed a wide array of consumptive multipredator effects, operating through a variety of mechanisms. When predator species hunt at different times and locations, this spatiotemporal complementarity can lead to more net prey consumption among co‐occurring predator species (Griffin, Byrnes, & Cardinale, 2013; Ives, Cardinale, & Snyder, 2005; Letourneau, Jedlicka, Bothwell, & Moreno, 2009). Indeed, prey escaping from predators in one spatial niche sometimes fall victim to a second predator species foraging elsewhere, leading to predator–predator facilitation (Losey & Denno, 1998; Soluk & Collins, 1988). In other cases, however, particularly voracious or effective single predator species may drive increased prey consumption within species‐rich predator communities, simply because diverse communities are more likely (by chance alone) to include functionally unique predators (O'Connor, Grabowski, Ladwig, & Bruno, 2008; Straub & Snyder, 2006). Likewise, especially large or aggressive predators sometimes act to disrupt feeding by other predators, relaxing net prey consumption (e.g., Finke & Denno, 2004). An extreme example of such strong species‐identity effects is seen with so‐called “keystone” predators (*sensu* Paine, 1969), which so effectively kill other abundant or impactful community members that they exert wide‐reaching changes in community composition and function (Menge, Berlow, Blanchette, Navarrete, & Yamada, 1994).

Of course, predators impact their prey not only by killing them, but also by inducing a range of behavioral, morphological, and physiological defenses that are energetically costly and can affect prey growth, survival, and reproduction (Werner & Peacor, 2003). The community‐level consequences of these “nonconsumptive effects” (NCEs) are well‐documented (Creel & Christianson, 2008; Peacor & Werner, 2001; Preisser, Bolnick, & Benard, 2005) and often equal or surpass those resulting from actual predation (Schmitz, Krivan, & Ovadia, 2004). As with consumptive effects, it can be challenging to predict the summed impact of several predator species' NCEs. In some cases, predators initiate trade‐offs between different and conflicting antipredator defenses, such that prey struggle to defend against two predator species at once (e.g., Bourdeau, 2009; Hoverman & Relyea, 2009; Ramirez & Snyder, 2009; Teplitsky, Plenet, & Joly, 2004); this can produce a net impact on prey analogous to consumptive complementarity. In other cases, strong predator species identity effects may prevail. For example, prey may scale the intensity of their defenses to reflect the “risk” posed by the most‐dangerous single predator species, indirectly also providing protection against less‐dangerous species (e.g., Huang & Sih, 1991; Eklöv, 2000; Laforsch & Tollrian, 2004; Steffan & Snyder, 2010). Elaborate experimental conditions often are needed to isolate NCEs from consumptive predator effects, and this has necessarily limited the diversity of predator species included in many NCE studies (Hoverman & Relyea, 2015), and sometimes precluded examination of behaviorally mediated predator–predator interactions that likely impact community‐wide NCEs (Relyea, 2003). While the full diversity of NCEs induced by multiple interacting predators is likely to be important within real‐world communities, these have yet to be fully explored (Kishida, Trussell, & Nishimura, 2009; Peckarsky & McIntosh, 1998).

**Figure 1:**
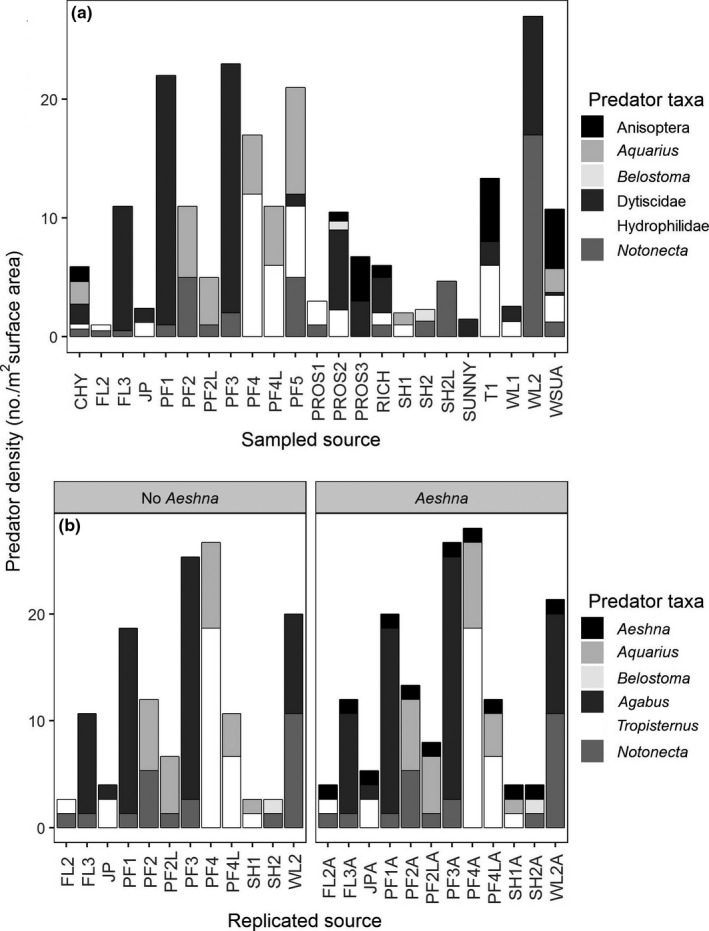
(a) Field surveys of predators attacking larval *Culex pipiens* larvae revealed most communities contained only two predator species and that dragonfly naiads (Anisoptera) generally appeared only in communities with more than two species. We designed an artificial‐pond experiment (b) with treatments that replicated the predator composition and density of each surveyed two species community (No *Aeshna*). Each of these mesopredator communities was replicated again with the addition of one *Aeshna* dragonfly naiad (*Aeshna*), yielding 24 unique predator communities

Here, we investigate NCEs that a diverse community of larval predators exerts on developing mosquito (*Culex pipiens*) larvae (Figure 1a). The mosquitoes face attack by this community of predators only as aquatic larvae, but we tracked the impact of the predators' NCEs to mosquito adults. Adult *C. pipiens* are vectors of several impactful vertebrate pathogens such as West Nile virus (Hamer et al., 2008) and avian malaria parasites (Kimura, Darbro, & Harrington, 2010). The larvae use energetically costly “diving” behaviors to escape from aquatic predators (Futami, Sonye, Akweywa, Kaneko, & Minakawa, 2008). Deployment of predator defenses as larvae can reduce adult mosquito longevity (Costanzo, Muturi, & Alto, 2011; Roux et al., 2015) and suppress mosquito immune function (Op de Beeck, Janssens, & Stoks, 2016), which might be expected to limit or increase, respectively, mosquito vectorial capacity (Kambris et al., 2010; VanderWaal & Ezenwa, 2016). However, this work exposed larvae to a single predator species, so it remains unclear how NCEs scale with more realistic levels of predator diversity that occur in natural habitats (e.g., Medlock & Snow, 2008). We fill this knowledge gap by: (1) surveying aquatic predators co‐occurring with *Culex* larvae in eastern Washington state, USA, to inform predator community compositions in a subsequent outdoor mesocosm experiment; (2) reconstructing the observed variation in predator community structure in experimental mesocosms, while measuring resulting NCEs on mosquito larvae and then adults; (3) observing mosquito diving behavior in the presence of various combinations of aquatic predators.

## 2 METHODS

### 2.1 Regional survey of Culex‐associated predator communities

#### 2.1.1 Survey methodology

Our field sites in eastern and central Washington state, USA (Fig. S1, Supporting Information), occurred within a matrix of agricultural fields and *Pinus ponderosa* forest at higher elevations, or sagebrush (*Artemisia* spp.) communities at lower elevations (Daubenmire, 1970). Annual rainfall ranges from 19.5 to 42 cm, depending on elevation, with a summer drought that likely restricts *C. pipiens* reproduction to seasonal ponds, irrigation ditches, and other bodies that retain water into the insects' July‐August breeding season (Stage, Gjullin, & Yates, 1952). We located possible breeding sites by visually searching online satellite images ( https://www.google.com/maps), and by opportunistically identifying smaller puddles and ditches while field‐scouting the larger sites, from June–August in 2013 and 2014. At each site thus located we censused aquatic predator communities using 1‐m^2^ net sweeps (BioQuip 12 in. D‐net), and mosquito communities using a 350 ml mosquito dipper (Bioquip). In the field, we counted predators and identified them to family (or genus, if possible), and 4^th^‐instar mosquito larvae to genus (see Appendix S1 for further details). In our region, the range of *C. pipiens* broadly overlaps with *C. tarsalis* (Stage et al., 1952); thus, the predator communities we sampled are likely representative of those experienced by some combination of the two *Culex* species.

#### 2.1.2 Describing *Culex*‐predator communities

We surveyed 34 water bodies containing aquatic predators (Fig. S1). Of these, we found 24 instances where aquatic predators co‐occurred with *Culex* mosquitoes. Predator communities included mesopredators such as *Notonecta* spp. backswimmers (Hemiptera: Notonectidae), *Aquarius* spp. water striders (Hemiptera: Gerridae), *Belostoma* spp. water bugs (Hemiptera: Belostomatidae), water scavenger beetles (Coleoptera: Hydrophilidae), and diving beetles (Coleoptera: Dytiscidae) (Figure 1a). Sites exhibited broad variation in the density and specific pairings of these species. We identified several morphospecies of beetles in the field, but owing to the difficulty in reliably assigning these beetles to species under field conditions, we did not identify these predators further.

Relatively larger *Libellula*,* Anax,* and *Aeshna* spp. dragonfly naiads were found in six of 23 communities, generally when ≥three predator species were found (Figure 1a). Aeshnid dragonfly naiads, including *Anax* and *Aeshna*, often occupy the top predator position in fishless ponds and pools (McPeek, 1998). These are generalist predators, known to prey on both mosquito larvae (Quiroz‐Martínez & Rodríguez‐Castro, 2007) and mesopredators (McPeek, 1998). The mesopredators we surveyed vary in their hunting domains, hunting modes, and in their capacity to limit mosquito populations. *Notonecta* readily consume mosquito larvae and have been reported to decrease mosquito populations in the field (Quiroz‐Martínez & Rodríguez‐Castro, 2007). Aquatic beetles undergo ontogenetic functional shifts, where larvae are obligate predators but adults are more omnivorous; adult Hydrophilidae are generally omnivorous, but opportunistically predaceous (Merritt & Cummins, 1996), while adult Dytiscidae are generally predaceous but opportunistic scavengers (Culler, Ohba, & Crumrine, 2014). We found both life stages in our surveys; however, adults were more common during the majority of the *Culex* breeding season. Dytiscid and Hydrophilid larvae have been reported to readily consume mosquito larvae, but adults are not very efficient predators of mosquitoes (Shaalan & Canyon, 2009). *Aquarius* skate on the water surface and consume insects that become trapped there, but gut content evidence has shown they occasionally consume mosquito larvae (Medlock & Snow, 2008). Because *Aquarius* are highly active on the water surface, there may be frequent opportunities for them to disturb mosquito larvae resting there.

### 2.2 Quantifying NCEs of realistically structured predator communities

Our field survey noted two consistent components of real‐world predator community structure. First, most predator communities co‐occurring with *Culex* larvae contained two species of aquatic mesopredators drawn from a diverse pool of taxa (see Figure 1a). Second, only the more‐diverse communities of ≥three species regularly included relatively larger *Libellula, Aeshna,* or *Anax* spp. dragonfly (Odonata: Anisoptera) naiads (Figure 1a). We reproduced each of these aspects of real‐world predator community structure in outdoor artificial ponds, where we could present predator cues to mosquito larvae, while blocking actual predation and measuring resulting effects on mosquito larval development and the condition of emerging adults. Our experimental design featured reproductions of each of the 12 different two‐mesopredator species communities observed in our regional survey, matching the field density of each predator species as closely as possible (Figure 1b) and fully crossing each of these mesopredator community compositions with the presence versus absence of a single *Aeshna* dragonfly naiad (Figure 1b). This yielded 24 unique predator communities (12 mesopredator compositions × 2 levels of naiad presence/absence = 24) that varied in predator species identities, density, species richness, and evenness (Figure 1b), each replicated once. Although we occasionally found Dytiscid and Hydrophilidae larvae, we only used the adult stages in our experimental communities because larvae were rare at the height of the mosquito breeding season when we performed this experiment. Despite finding several morphospecies of aquatic beetles, we only used the most common member of each family in the mesocosms; this was *Agabus* spp. representing Dytiscidae and *Tropisternus* spp. representing Hydrophilidae.

Artificial ponds were plastic wading pools (0.91 m diameter × 35 cm deep), filled to a depth of 20 cm with dechlorinated tap water. Artificial ponds were housed on the Washington State University Campus in Pullman, WA USA, under a 60% shade cloth enclosure. During the experiment, the mean daily low temperature was 11.9°C (range 5.7–17.2°C), and the mean daily high temperature was 30.9°C (range 20.5–36.4°C). We inoculated each pool with 0.5 g of brewer's yeast as an initial food source for mosquito larvae (Costanzo et al., 2011), but mosquitoes also had access to naturally colonizing microorganisms as their primary food source. *Culex pipiens* are “collector‐filterers” that feed on microorganisms either suspended in the water column or collected on the surface of detritus (Yee, Kesavaraju, & Juliano, 2004).

Twenty‐four hours after inoculating ponds with yeast, on 16 July 2014, we placed a cage in each pond constructed from a 35.5 × 23 cm plastic tub with holes drilled in the sides covered with 0.3 mm fiberglass mesh, allowing water, microorganisms and chemical cues to exchange between the two arenas (Fig. S2). The main arena held predators and mosquito larvae that were exposed to predation, while the cage held a subset of larvae that were protected from predation. Next, we released 200 one‐day‐old *C. pipiens* larvae in the main arena and 50 larvae in the cage. *Culex pipiens* larvae used in this and subsequent experiments were from a laboratory colony founded in the summer of 2013 and maintained by the authors at Washington State University Pullman, WA USA. Eight hours later, we released predators into the main arena; these predators were collected from water sources surrounding Pullman, WA, USA, then stored in an incubator, without access to food, at 23°C for 24–48 hr before being released into our experimental arenas. Intraguild predation was rare (*N *=* *2 confirmed events), but predators occasionally turned up missing or dead from unknown causes. To preserve predator community structure throughout the experiment, we replaced any missing, dead, or killed predators within 24 hr. We also constructed, in the absence of predators, 4 ponds with 100 exposed/50 caged larvae, and four ponds with 200 exposed/50 caged larvae, such that total *N *=* *32 replicate ponds across the entire experiment. We chose two densities for control conditions because we assumed that predators would reduce the density of exposed mosquito larvae in the predator treatments, which could have density‐mediated effects on mosquito condition. The 100‐larvae control would represent the density reduction caused by predators, without any possible fear effects induced by their presence. Although we included no‐predator controls in this experiment, our goal was not to compare the traits of mosquitoes emerging from predator‐free environments with those reared in the presence of predators. Rather, our experiment was designed to test: (1) if mosquito traits were correlated with aspects of predator community composition and evenness and (2) if mosquito traits differed between the mesopredator and mesopredator + *Aeshna* communities.

Every day after predator release, we checked the artificial ponds to (1) count the number of remaining mosquito larvae in and outside of the cages, (2) replace any predators that had died or escaped, (3) remove any mosquito eggs that were deposited during the previous night, and (4) collect any mosquito pupae. Collected pupae were placed into plastic cups (~5 cm diameter) containing 10 ml dechlorinated water, with ≤five pupae per cup, and maintained in the laboratory (14:10 light cycle, 22–24°C) in 0.5‐L (7.6 cm diameter × 8.5 cm tall) cardboard containers. Each day, we checked the cardboard containers for mosquitoes that had eclosed to the adult stage. Upon emergence of the first adults, we added a 2‐dram glass vial containing a 3% sucrose solution, wicked with a piece of cotton (to serve as a food source). We checked adult mosquitoes daily until death, for up to 50 days (when 95% of the mosquitoes had died). Upon death, we measured adult wing length as an indicator of adult body size (Packer & Corbet, 1989), by removing, slide‐mounting, and photographing wings using a Leica EZ4 stereo microscope. Then, we measured the distance from the distal margin of the alula to the distal tip of the R3 vein using ImageJ software (National Institutes of Health, Bethesda, MD, USA). Throughout this experiment, we collected data on mosquito larval development period, larval survival, adult longevity, and adult body size. These mosquito traits were chosen because they can contribute to the vectorial capacity and vector competence of mosquitoes (Roux et al., 2015).

### 2.3 Observing predator and prey behavior

Mosquito and predator behaviors were difficult to observe in the open‐field ponds and ditches, or in our artificial ponds. To gain insight into how mosquito larvae might alter their behavior when facing attack by different species and/or compositions of aquatic predators, and to document how community composition altered behavior of the predators themselves, we conducted timed observations in smaller arenas. Here, our experimental replicates were 25 × 37.5 cm plastic tubs (microcosms), placed on a laboratory bench (14:10 light cycle, 22–24°C). To these we added 4 L of dechlorinated tap water and 16 ml of larval diet slurry used in colony rearing (0.5 g of a 1:2:1 ratio of beef liver powder: rabbit chow: fish flakes per ml distilled water). All slurry ingredients were finely ground and well‐mixed into distilled water. Despite the short duration of this experiment, larval diet was added to microcosms so we could observe how predators influence larval feeding behaviors. However, we found that the minute movements of larval *C. pipiens'* mouthbrushes deployed during filter‐feeding were too difficult for us to reliably score during observational scans. Each microcosm included two 10 × 5 cm pieces of floating fiberglass window screen, and one submerged 1.25 cm diameter PVC elbow connector, to serve as mosquito refuges. Mosquitoes were exposed to either monoculture or polyculture predator treatments, or a no‐predator control. Monocultures consisted of two individuals of each mesopredator (*Aquarius*,* Agabus*,* or Notonecta*), while polycultures consisted of factorial combinations of each of the mesopredators with one *Aeshna* naiad. Each treatment was replicated four times, yielding a total of 28 experimental units (*N *=* *4 per predator species composition; total *N *=* *28). Prior to the start of the behavioral assays, predators were collected and treated as described in the mesocosm experiment.

We transferred 50 third‐instar *C. pipiens* larvae to each tub, allowing the larvae 1 hr to acclimate before adding predators. The mosquitoes used in this experiment were fed ad libitum, with the same larval diet mentioned above, until the start of the experiment. We waited an additional hour to begin observations after adding the predators. For this experiment, all predators and mosquitoes were in the same arena and were free to interact (i.e., no caging was deployed). We made four 1‐min‐long observations of each arena at 16:00, 18:00, 20:00, and 23:00; the last observation was made under a red light during the dark cycle. Before each behavioral observation, we counted the number of larvae remaining in the microcosm, allowing time for larvae to acclimate to our presence. During each observation, we recorded the proportion of larvae moving laterally versus diving, and if non‐*Aeshna* predators were active (any movement that propelled the predator more than ~1 cm). We averaged responses across the four observation periods for analyzes.

### 2.4 Statistical analyzes

For data from the artificial pond experiment, we first used the R package MuMin (Bartoń, 2016) to perform model selection (using Akaike information criterion corrected for small sample size, “AIC_c_”) to evaluate NCEs on larval mosquito survival and development time, and adult body size and longevity. Factors considered in our full model were the density of each predator taxon (but we lumped *Agabus* and *Tropisternus* aquatic beetles into a “beetle” category to reduce the number of terms), predator evenness (as measured by Shannon's equitability, E_*H*_), and the presence/absence of an *Aeshna* naiad. We also considered possible interactions between *Aeshna* presence/absence and predator evenness and density in the full model. The larval survival data fit a quasibinomial error term, and thus, we ranked models for this response variable using quasi‐AIC (QAIC) because quasi‐models do not report a likelihood (Bolker, 2016). As we were interested in the effects of predator community attributes (many of which were continuous variables) on mosquito traits, we excluded the control replicates from these analyzes. In each case, we selected the best model based on a combination of lowest AIC_c_ score and fewest parameters (Burnham & Anderson, 2002). For cases when the null model scored highest, we still ran statistical tests on the next‐best model with fewest parameters. For each dependent variable except larval survival, we performed multiple linear regression to test the relationship between the predator community attributes indicated in the top model(s) and the response variable. We used pooled caged and uncaged mosquitoes for these analyzes because we found no significant differences among exposure treatments for adult longevity (Fig. S3C) or adult body size (Fig. S3D). There was a statistically significant difference in larval development period between exposure treatments (*df* = 42, *F *=* *4.291, *p *=* *.0445; Fig. S3B), but we regarded the biological significance minute (14.7 vs. 15.2 days for caged and uncaged mosquitoes, respectively) and still performed analyzes on pooled mosquitoes. There were significant differences in survival of caged versus exposed larvae (Fig. S3A), so we only considered caged mosquito larvae in the model. Because of sexual dimorphisms, we sex‐standardized mosquito adult longevity and wing length before analysis (Figs S4C,D; see Appendix S1 for methods). Although not the primary focus of our study, we also include a comparison of each predator treatment (mesopredator & mesopredator + *Aeshna*) with controls in Fig. S4). In our behavioral observation trial, our statistical model included the factors mesopredator species composition, *Aeshna* present/absence, and their interactions. Mosquito behavior (proportion diving or active) was analyzed using a glm with binomial errors weighted by the number of larvae in each pond (mosquito numbers declined through time as they were eaten by predators; Fig. S5). For analyzes of predator activity, we used a glm with quasibinomial errors. All analyzes were performed using R version 3.1.1 (R Core Team, 2014).

## 3 RESULTS

### 3.1 NCEs of realistically constructed predator communities

**Figure 2:**
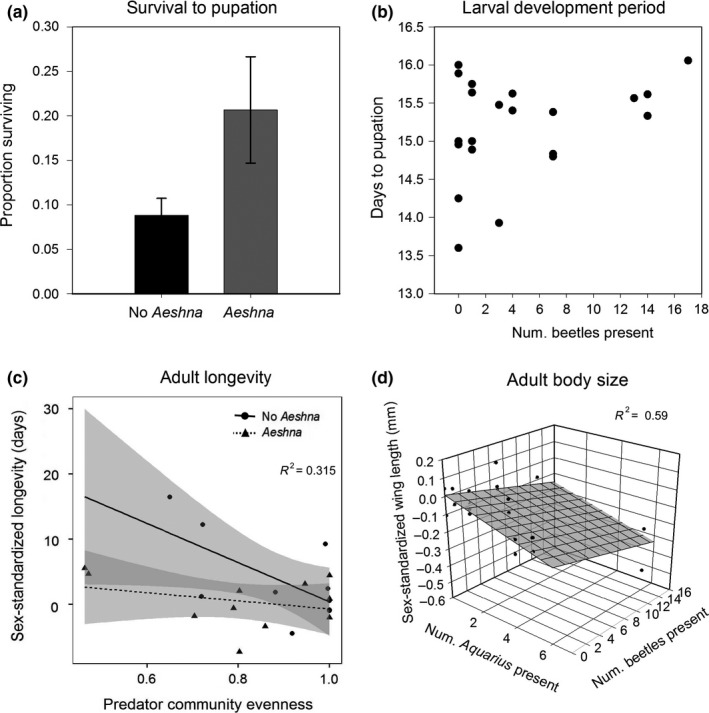
The influence of larval predator community attributes selected in Table 1 to best predict *Culex pipiens* (a) larval survival, (b) development time, (c) adult life span, and (d) adult body size. For panel a, bars are group means and error bars are mean ± SE. Different letters indicate statistical difference of means. For panels b‐d, each data point represents the mean of mosquitoes reared in a community. In panels c and d showing sex‐standardized trait values, points falling above zero mean individuals on average had greater trait values than those for their respective sex in the control scenario; those below zero indicate lower trait values (Appendix S1). Lines were only included if there was a significant relationship with the predictor variables. Statistics are presented in Table 2

We are reporting the results of analyzes performed on the top models, as indicated by AIC_c_ model selection criterion (Table 1). QAIC model selection indicated that *Aeshna* presence/absence and *Notonecta* density were both candidate factors describing *Culex* survival through larval development (Table 1). Caged mosquitoes, exposed to cues associated with predators and predation of mosquitoes, were half as likely to survive to pupation when in the presence of mesopredators, across all community compositions, than when dragonflies were also present alongside the mesopredators (Figure 2a, Table 1). Additionally, there was a positive relationship between *Notonecta* density and the proportion of mosquitoes that survived through larval development (*df* = 22, *F *=* *9.443, *p *=* *.0056; Fig. S6). Examination of Fig. S6 reveals two possible influential points that could be driving this relationship, and subsequent Cook's distance analysis revealed these two points have large distance values (>1). Therefore, we consider these results inconclusive at this point.

**Table 1:** The three best performing (i.e., lowest Akaike information criterion corrected for small sample size, AIC_c_) models are listed with the chosen model shown in bold face. For each model, values are shown for the estimated number of model parameters (*k*), maximum log‐likelihood (LL) or quasi‐likelihood (QL), the information‐theoretic Akaike's information criterion corrected for small sample size (AIC_c_), the change in AICc relative to the top‐ranked model (ΔAIC_c_), and the Akaike weight (*w*_*i*_)

*Culex pipiens* response	Model	*k*	QL	QAIC	ΔQAIC	*w*_*i*_
1) Survival	Notonectidae	2	−4.287	12.6	0	0.296
	Null	1	−5.584	13.2	0.59	0.220
	***Aeshna***	**2**	−**3.875**	**13.7**	**1.18**	**0.165**
*C. pipiens* response	Model	*k*	LL	AIC_c_	ΔAIC_c_	*w*_*i*_
2) Development	Null	1	−20.436	45.5	0	0.199
	**Beetles**	**2**	−**19.213**	**45.8**	**0.30**	**0.172**
	Beetles + Evenness	3	−18.667	47.8	2.30	0.063
3) Longevity	*Aeshna* + Evenness	3	−61.930	134.4	1.45	0.089
	***Aeshna *** **× Evenness**	**4**	−**60.222**	**134.4**	**1.54**	**0.085**
	(*Aquarius* + Beetle) × *Aeshna* + Evenness	7	−69.985	168	0	0.246
4) Wing length	(*Aquarius* + Beetle) × *Aeshna*	6	−73.047	168.7	0.74	0.170
	***Aquarius*** + **Beetle**	**3**	−**79.375**	**169.3**	**1.28**	**0.129**

Three of the 24 experimental units containing predators produced no mosquito pupae or adults to measure, therefore, for the remaining results from this experiment, *N *=* *21. The length of the larval development period was not significantly correlated with any predator community attribute (Figure 2b; Table 2). We chose to analyze the *Aeshna* × evenness interaction model to describe adult mosquito longevity, based on a combination of its low AIC_c_ score and relatively fewer number of parameters (Table 1). For adult mosquitoes, increasing predator community evenness correlated with decreasing survivorship, however, the presence of dragonfly naiads further reduced adult mosquito longevity across levels of predator community evenness (Figure 2c, Table 2). We chose to analyze the *Aquarius* + Beetle model to describe adult mosquito body size based on a combination of low AIC_c_ score and relatively fewer number of parameters compared to other top models indicated by AIC_c_ criteria alone (Table 1). Protected (caged) larvae developed into smaller adults when placed with higher densities of predatory beetles and *Notonecta* (Figure 2d, Table 2). These NCEs were independent and additive.

**Table 2:** The effects of predator community attributes kept in the best model for *Culex pipiens* larval survival (A), larval development period (B), adult longevity (C), and adult body size (D). Results were analyzed using linear regression. Significant effects are indicated in bold

Response	Model	Factor	Estimate	SE	*t*	*F*	*p*
A) Development	Beetles					2.348_(1,19)_	.142
		Beetles	0.0396	0.026	1.532		.142
B) Survival	*Aeshna*					4.339_(1,23)_	**.049**
		*Aeshna*	0.9890	0.493	2.004		**.058**
C) Longevity	*Aeshna* × Evenness					3.373_(3,17)_	**.0429**
		*Aeshna*	−24.90	12.02	−2.072		**.054**
		Evenness	−30.02	11.35	−2.645		**.017**
		*Aeshna* × Evenness	23.79	13.73	1.733		.101
D) Adult size	*Aquarius* + Beetles					14.31_(2,18)_	**<.01**
		*Aquarius*	−4.5346	1.135	−3.993		**<.01**
		Beetles	−1.5221	0.468	−3.254		**<.01**

### 3.2 Predator/prey behavior assay

**Figure 3:**
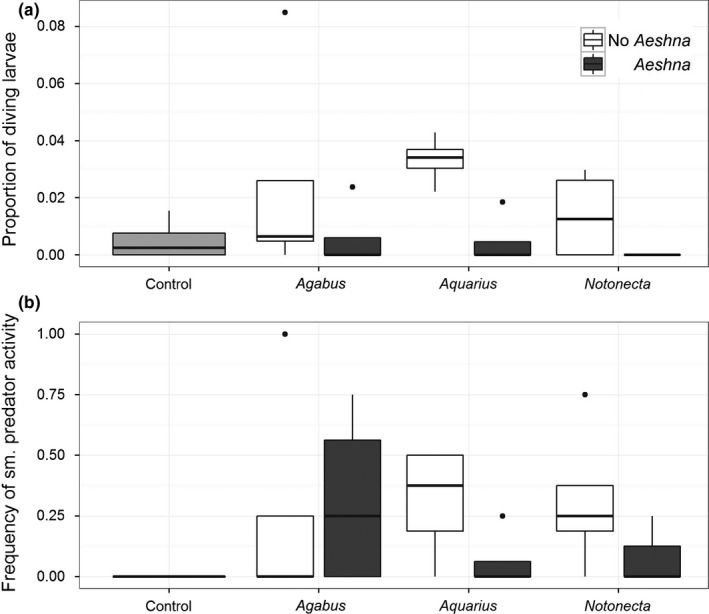
Data from laboratory behavioral bioassays testing the survival and behavioral effects of predator exposure. Box plots show the distribution of the proportion of larvae observed diving and (a) the frequency of mesopredator activity (b). Treatment groups include a no‐predator control (light gray box plot) and larvae exposed to the following predator taxa: *Aquarius* water striders, *Agabus* diving beetles, and *Notonecta* backswimmers alone (white box plot) or with an *Aeshna* nymph (dark gray box plot)

In arenas where predators and *C. pipiens* larvae could freely interact (i.e., predation was not prevented by caging), mosquito larvae regularly deployed “diving” escape behavior in the presence of the mesopredators alone, but diving was dramatically reduced when a dragonfly naiad was present alongside the mesopredator (*Aeshna* main effect: *df* = 19, *Wald* χ^2^ = 14.657, *p *=* *.04158; Figure 3a). Predator activity was consistent across predator species (Predator species main effect: *df* = 20, *F *=* *1.5880, *p *=* *.2271; Figure 3b), and not altered by *Aeshna* (Treatment × *Aeshna: df* = 18, *F *=* *1.4518, *p *=* *.2438; *Aeshna* main effect, *df* = 19, *F *=* *0.4013, *p *=* *.5344).

## 4 DISCUSSION

Nonconsumptive effects strongly influence the impacts of predators on their communities (Preisser et al., 2005; Schmitz et al., 2004; Werner & Peacor, 2003), but it is unclear how NCEs scale as communities become more species‐rich. For predators' consumptive effects, we know that opportunities for complementarity (e.g., Gable, Crowder, Northfield, Steffan, & Snyder, 2012), facilitation (e.g., Losey & Denno, 1998), and interference (e.g., Finke & Denno, 2004) among predator species all increase with greater predator species richness; the balance among these sometimes‐opposing interactions determine whether herbivore suppression strengthens or weakens at higher diversity levels (Griffin et al., 2013; Ives et al., 2005; Letourneau et al., 2009). We considered the possibility that a rich array of NCEs might occur among the community of aquatic predators attacking mosquito larvae. A regional survey of seasonal water bodies occupied by larval *Culex* spp. revealed a range of predator relative densities and species compositions (Figure 1). Despite this compositional diversity, two broad patterns emerged. First, the modal condition was two species of predatory bugs or beetles, although the identities of those two species, and their relative densities, varied broadly (Figure 1). Second, dragonfly naiads, the top predator in these communities, were usually found in communities including >two other predator species (see also McPeek, 1998; McCauley, 2007; Kishida et al., 2009; Figure 1).

We recreated this natural variation in predator evenness and species identity in artificial ponds, in the presence versus absence of a single *Aeshna* dragonfly naiad. By protecting a subset of *C. pipiens* larvae from predation but allowing exposure to predators' chemical cues, we were able to examine factorial impacts of both factors on NCEs impacting mosquitoes. We found a diverse array of NCEs that sometimes exerted effects spanning mosquito developmental stages. During the larval stage, we detected lower survival in mosquitoes exposed to mesopredator communities than those exposed to mesopredator + *Aeshna* communities (Figure 2a). This suggests three nonmutually exclusive mechanisms could be at play. The first is that *Aeshna* + mesopredator communities consumed more exposed larvae, which freed resources for remaining caged larvae. However, we found no differences in survival among exposed mosquitoes reared in the two types of predator communities (Fig. S4). The second is that antipredator defenses in response to *Aeshna* might be conflicting with those initiated by mesopredators. If mosquitoes respond to this scenario by either forgoing predator defenses or hierarchically responding to *Aeshna*, a similar increase in survival could result if *Aeshna*‐induced defenses are less costly. A third possibility is that *Aeshna* alters the behavior of mesopredators, which in turn modifies the amount of risk perceived by their shared prey (e.g., Kishida et al., 2009).

Our behavioral assay data provided some support for a combination of the latter two mechanisms. We regularly observed *C. pipiens* larvae deploying diving behavior in response to the mesopredator species. Diving is a well‐known predator‐escape behavior for these insects that have been described primarily as a mechanism to avoid surface (e.g., *Aquarius*) and aerial predators (e.g., Futami et al., 2008; Figure 3a), but we observed the behavior in response to sudden movements by any of the mesopredators. However, when the top predator *Aeshna* naiad was also present, this defense behavior was rarely deployed (Figure 3a). *Aeshna* naiads are sit‐and‐wait predators that occupy the lower water column, so diving behaviors normally practiced by mosquitoes to escape the mesopredators could make mosquito prey more conspicuous and susceptible to *Aeshna* predation. Mosquitoes may have hierarchically responded to the presence of *Aeshna* and abstained from diving to reduce the probability of encountering this risky, bottom‐dwelling predator. Indeed, we observed lower survival over the 24‐hr observation period in the treatments that included *Aeshna* than in the mesopredator‐only treatments (Fig. S5), but we note these observations were performed in smaller arenas than the mesocosm experiment which likely increased contact rates among mosquitoes and *Aeshna*. We also observed that two of the mesopredator species (*Aquarius* sp. and *Notonecta* sp.) tended to reduce their activity in the presence of the top predator, but *Agabus* beetles exhibited variable behavior, and as such no overall‐significant effect of *Aeshna*'s presence was detected (Figure 3b). It is possible then that the top predator is triggering a behavioral change in some mesopredator species, as has been commonly reported in other study systems (e.g., Crumrine & Crowley, 2003; Kishida et al., 2009; Moran, Rooney, & Hurd, 1996). We did not observe mosquito responses to *Aeshna* in isolation because we never observed this condition in the field, so we cannot determine if the reduction in diving observed between mesopredator and mesopredator + *Aeshna* communities is due to a hierarchical response to *Aehsna* predation risk or if it is mediated by changes in mesopredator activity. These two possibilities are not mutually exclusive; however, and both may be acting to produce the results we observed.

Adult *Culex* mosquitoes emerge from their aquatic larval habitat to terrestrial environments, where female mosquitoes notoriously vector pathogens such as West Nile virus. Larval stage stresses have been shown to have lasting effects in many organisms with complex lifecycles, including mosquitoes (Roux et al., 2015). Therefore, we tracked the traits of adult mosquitoes emerging from our mesocosms to observe if differences in larval predator community structure have lasting effects on adult traits pertinent to pathogen transmission cycles. Increasing predator community evenness was negatively correlated with longevity for adult mosquitoes that emerged after being exposed to predator cues as larvae (Figure 2c). We are unaware of studies that examine the effects of predator evenness on NCEs; however, recent studies have shown that greater natural enemy evenness can enhance prey suppression in agricultural settings (e.g., Crowder, Northfield, Strand, & Snyder, 2009). One way evenness could influence NCEs is if the relative abundance of predators plays a role in how prey choose to respond to them. For example, prey may face a greater challenge balancing defenses against equally abundant predators than if one predator is rare and another is abundant, in which case prey may choose to defend against the more abundant predator. Although we are unsure of the mechanism, it appears greater predator community evenness may have caused additional stress to developing mosquito larvae, which resulted in less robust adults. Furthermore, across levels of predator evenness, larval‐stage exposure to *Aeshna* naiads further reduced adult mosquito longevity (Figure 2c). Although the presence of *Aeshna* during the larval stage conferred a benefit to caged mosquito larvae (i.e., greater survival through larval development), there appears to have been a tradeoff where defenses useful in one life stage harm the condition of prey in subsequent life stages (e.g., Skelly & Werner, 1990; Walsh, Downie, & Monaghan, 2008). We found further lifestage‐spanning NCEs when we measured adult mosquito wing length. Increasing densities of predatory beetles correlated with smaller mosquito adult sizes (individual *R* ^2^ = 0.23; Figure 2d); increasing densities of the water strider *Aquarius* spp. triggered an additional, additive and independent NCE that likewise decreased adult mosquito size (individual *R* ^2^ = 0.36; Figure 2d).

In summary, we found many instances where the addition of a top predator to mesopredator communities altered the NCEs imposed by mesopredators. The impact of the top predator was seen across variation in mesopredator density and community structure. Because the addition of a single *Aeshn*a naiad acted to suppress mosquito diving behavior, increased mosquito survival through larval development, and subsequently decreased adult mosquito lifespan, this top predator has a unique, and uniquely strong, impact on community‐wide NCEs. This suggests a phenomenon roughly analogous to the consumptive impacts of “keystone” predators (*sensu* Paine, 1969; see also Valls, Coll, & Christensen, 2015), where relatively small numbers of individuals, through their feeding on other species, have disproportionate impacts that radiate out to impact the community as a whole. Indeed, the prototypical keystone predator, the seastar *Pisaster ochraceus*, impacts other community members at least in part through nonconsumptive means (Morgan, Gravem, Lipus, Grabiel, & Miner, 2016). Our findings complement several other recent studies demonstrating that NCEs exerted by species‐rich predator communities reflect mechanisms analogous to those leading to predator diversity effects through consumptive channels (e.g., Hatcher, Dick, & Dunn, 2014). For example, energetically costly predator‐avoidance behaviors that drain physiological resources of prey may weaken the prey's ability to mount an effective immune response against pathogens (e.g., Ramirez & Snyder, 2009); this is a form of NCE‐mediated facilitation among natural enemies. Likewise, defenses that require prey traits to move in one direction to protect against one predator species, but another direction to defend against a second predator, can lead to intermediate strategies/morphologies not fully protective against either predator species (e.g., McIntosh & Peckarsky, 1999). Our results support the argument that recreating natural variation in predator biodiversity, and thereby portraying the broad ranges of predator community compositions typical of many field situations, provides an opportunity to capture the full diversity of multipredator effects through nonconsumptive mechanisms (Calcagno, Sun, Schmitz, & Loreau, 2011; Davenport & Chalcraft, 2013; Hoverman & Relyea, 2015). Although we believe it is clear *Aeshna* is altering NCEs imposed by mesopredator communities, some of the patterns we observed (e.g., increased *C. pipiens* larval survival, but decreased adult longevity in *Aeshna* communities) are difficult to describe mechanistically with our design, which did not take any physiological measurements such as respiration rate or stress hormone measurements. We sacrificed replication of predator communities for inclusion of many communities in the mesocosm experiment, but see our study as a vital first step in recognizing the importance and diversity of multipredator effects through nonconsumptive mechanisms.

Our study joins a number of others (e.g., Benard, 2004; Davenport, Hossack, & Lowe, 2014; De Block & Stoks, 2005; Vonesh, 2005) reporting NCEs exerted on one prey life stage that continue to impact later life stages. For prey species that undergo dramatic metamorphoses, this means that NCEs on early stages can have effects that radiate into very different habitats and ecological contexts (e.g., Ficetola & De Bernardi, 2006). Such stage‐bridging NCEs could be of particular interest for prey that acts as pathogen vectors, such as the mosquitoes that we considered (Costanzo et al., 2011; Roux et al., 2015). While predators that kill vectors might obviously dampen transmission (e.g., Moore, Borer, & Hosseini, 2010), NCEs exerted on vectors may have more subtle effects on disease dynamics (Finke, 2012). For example, our study found that particular predator combinations altered mosquito longevity by 5 days or more (Figure 2c). Extrinsic incubation periods for many pathogens that impact humans (e.g., malaria parasites, West Nile virus, and Dengue virus) often are relatively long when compared to the life spans of their mosquito vectors (Bara, Rapti, Cáceres, & Muturi, 2015). This means that relatively modest increases or decreases in vector life spans can have fairly dramatic impacts on whether vectors regularly live long enough to transmit pathogens to new hosts (e.g., LaDeau, Allan, Leisnham, & Levy, 2015; McMeniman et al., 2009; Shapiro, Murdock, Jacobs, Thomas, & Thomas, 2016). For example, *C. pipiens* requires ~14 days between becoming infected from a blood meal and being able to secondarily transmit West Nile virus (e.g., Anderson, Main, Delroux, & Fikrig, 2008). Our results suggest that nonconsumptive predator effects could be an underappreciated means for predators to indirectly alter pathogen transmission by vector–prey that escape being killed.

## AUTHOR CONTRIBUTIONS

AJM, JPO, and WES conceived the ideas and designed methodology; AJM collected the data; AJM analyzed the data; AJM and WES led the writing of the manuscript. All authors contributed critically to the drafts and gave final approval for publication.

## DATA ACCESSIBILITY

DRYAD entry doi:10.5061/dryad.5j329.

## CONFLICT OF INTEREST

None declared.
