# Supplementary material for: Keystone nonconsumptive effects within a diverse predator community
Source: Ecol Evol. 2017 Oct 28;7(23):10315–25. doi: 10.1002/ece3.3392 (PMC5723625; doi:10.1002/ece3.3392)
Supplement: Supplementary file 1 [file ECE3-7-10315-s001.docx]

**Fig. S1.** Sampling locations of predator community surveys conducted over the 2013 & 2014 growing seasons. Gray circles represent locations where both *Culex* mosquito larvae and predators were collected, while black circles represent locations where only mosquitoes *or* predators were collected. Sampling sites are jittered on the map projection so that nearby sites do not obscure one another.


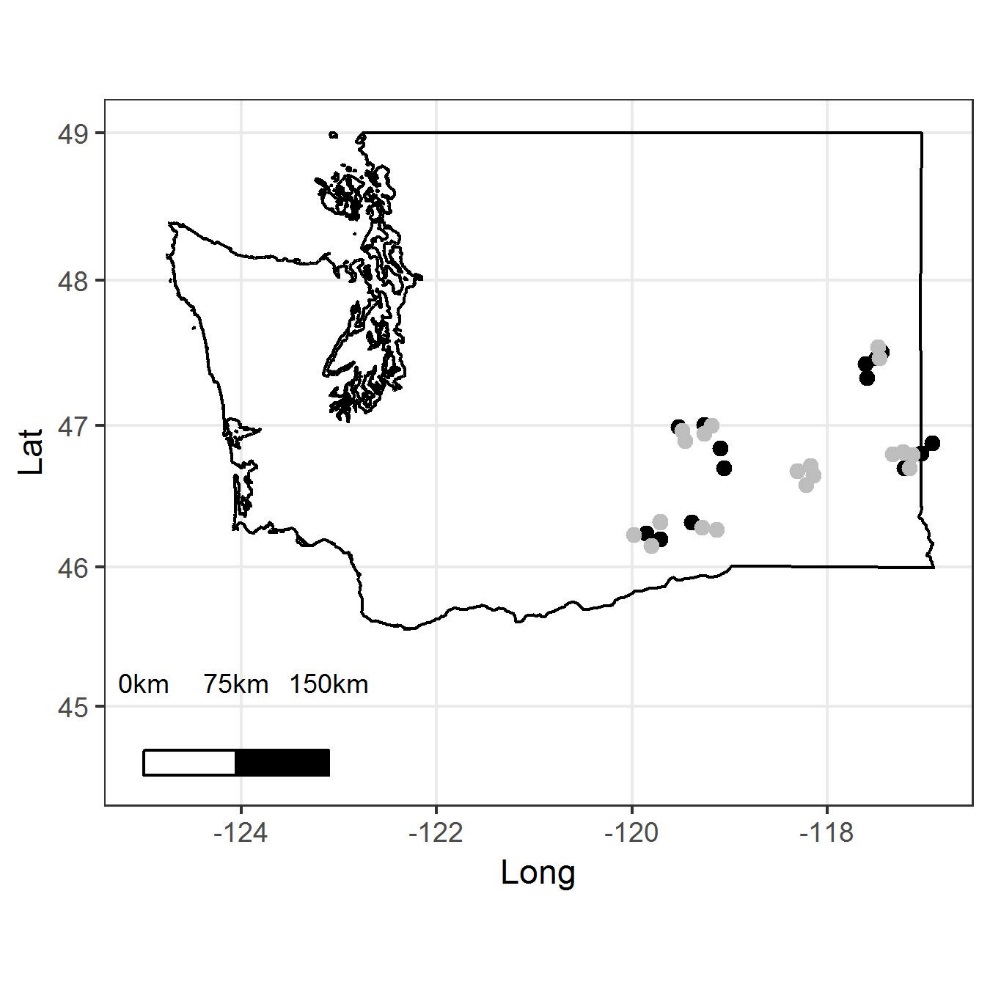


**Fig. S2.** An artificial pond, showing the mesh cage that protected a subset of developing mosquito larvae from predation.


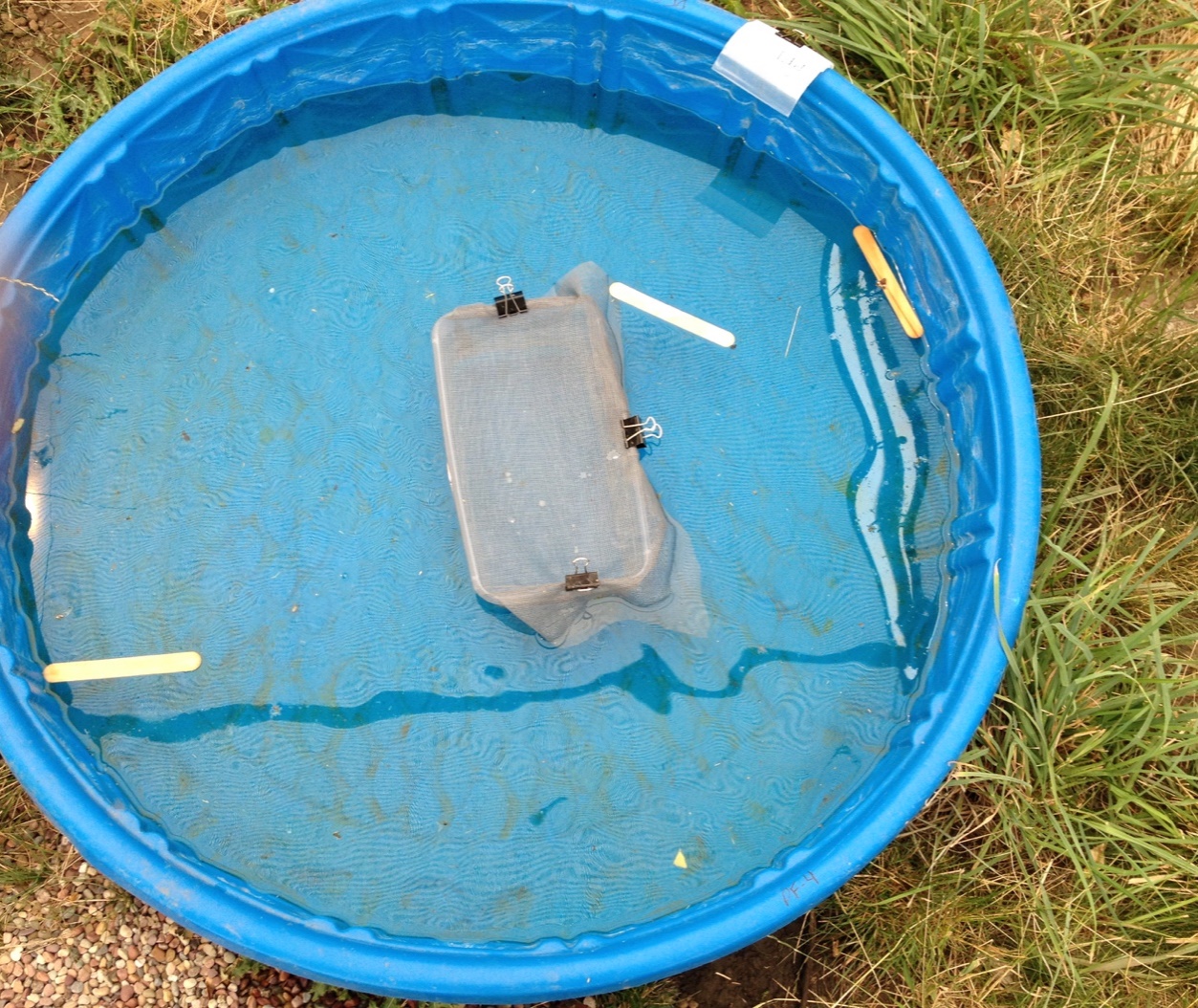


**Fig. S3.** The difference between caged (“C”) and uncaged (“NC”) mosquito traits from the artificial pond experiment, for (A) survival and (B) larval development time of *Culex pipiens* larvae, and (C) adult longevity and (D) wing length of adult male and female mosquitoes. We were unable to compare male and female survival to pupation, because we could not discern the sex of larval mosquitoes. Data are means ± SE. Means differed significantly among caged and exposed mosquitoes for (A) larval survival (*t* = 2.596, *df* = 33.453, *P* = 0.014) and (B) larval development period (*t* = -2.3221, *df* = 20.084, *P* = 0.031).


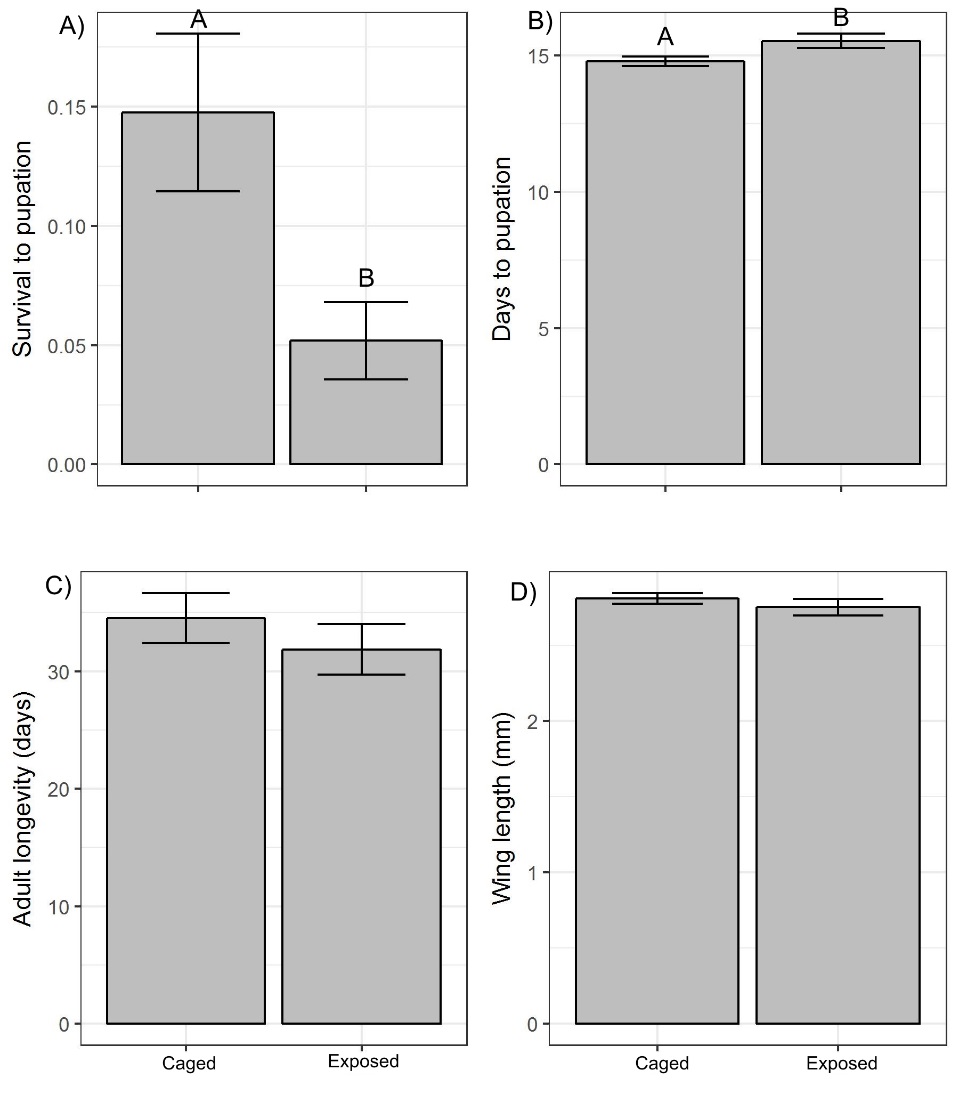


**Fig. S4.** Mean trait values for mosquitoes reared in no-predator control, mesopredator, and mesopredator + *Aeshna* treatments. Traits measured include: (A) survival to pupation of caged and exposed mosquitoes, (B) duration of larval development, (C) adult longevity, and (D) adult wing length. Data are means ± SE. Different letters over bars signify a significant difference significantly among predator treatments. Asterisk indicate a significant difference in trait values among sexes for a particular treatment group. Survival to pupation and development period data were analysed with a one-way ANOVA; Longevity and wing length data were analysed with a two-way ANOVA.
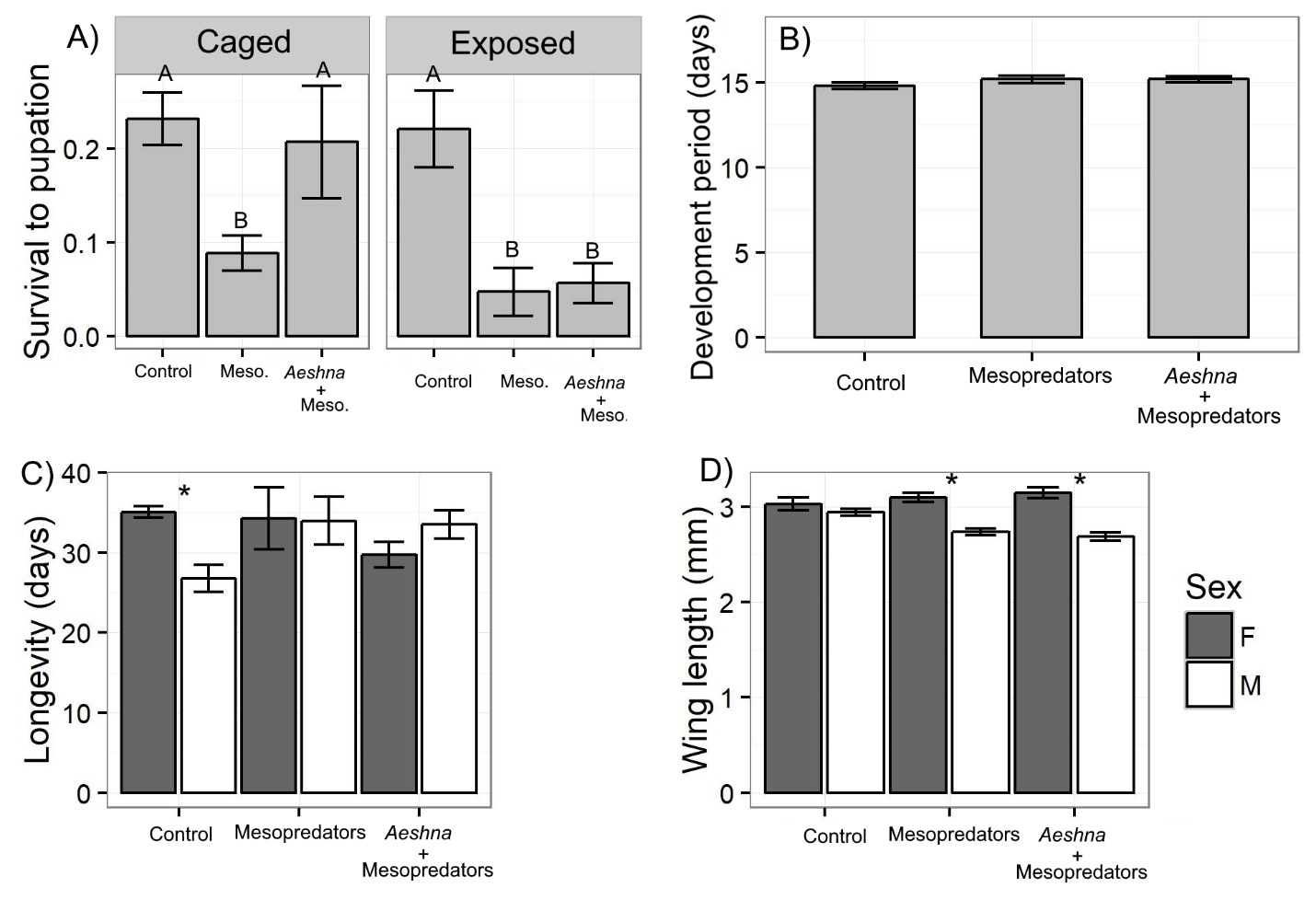


**Fig. S5.** Data showing larval *C. pipiens* survival throughout laboratory behavioral bioassays where mosquitoes were not protected from predation with a cage. Box plots show the distribution of the proportion of surviving larvae over the four observation periods. Treatment groups include larvae exposed to the following predator taxa: *Aquarius* water striders, *Agabus* diving beetles, and *Notonecta* backswimmers alone (dark gray box plot) or with an *Aeshna* naiad (white box plot). There was a significant Treatment × *Aeshna* interaction (*df*= 15, χ^2^= 182.76, *P*= 0.0434) for survival; substituting *Aeshna* for one of the small predators generally decreased mosquito survival, but this trend was not consistent among all of the small predator identities.


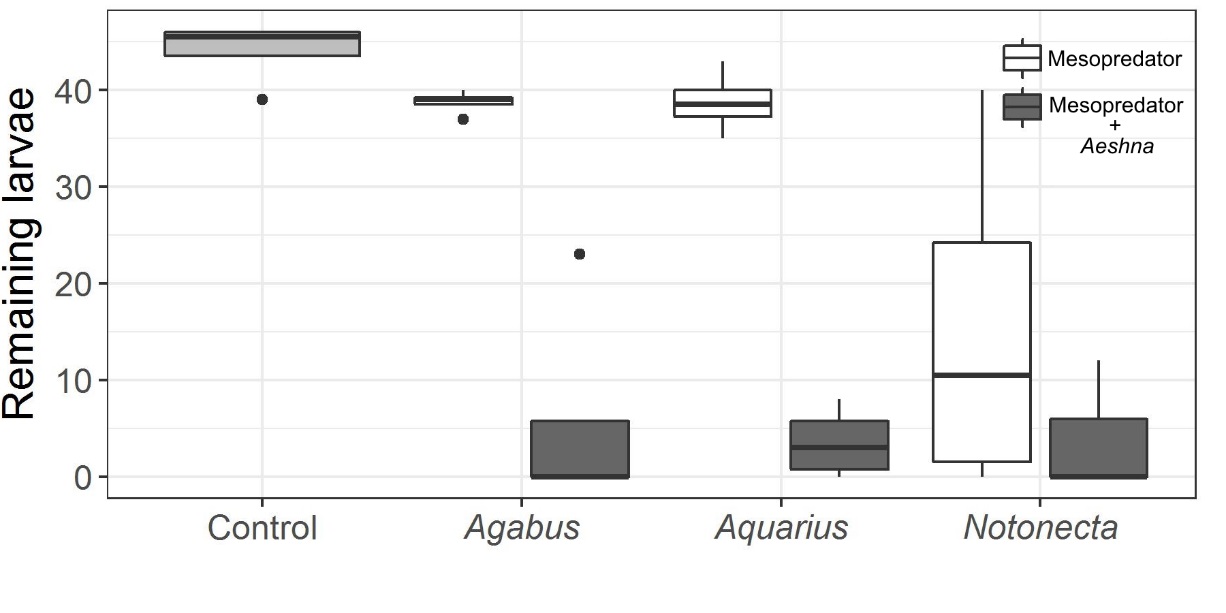


**Fig. S6.** The positive relationship between the number of *Notonecta* present in experimental mesocosms and the proportion of caged mosquito larvae, exposed to predator cues, that survived to their pupal stage (*df* = 22, *F* = 9.443, *P* = 0.0056).


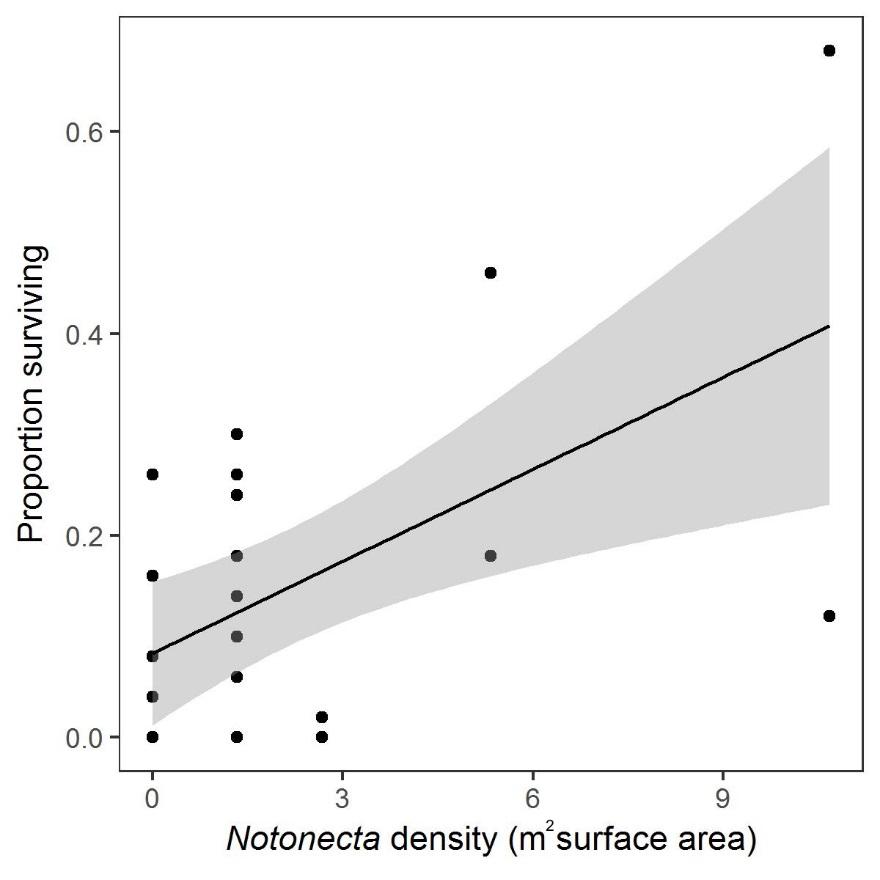


**Appendix S1**

Methods related to open-field sampling and the artificial-pond experiment.

*Details of open-field sampling*

Three water sources that were located early in the growing season were permanent enough to show temporal differences in predator communities, so we included an “early” and “late” community from each of these sites. During each visit to each water body, we performed several 1 m^2^ D-net sweeps around the perimeter of each water source. Because water bodies varied in size from small puddles to large marshes, we used a random list of numbers between 1 and 5 to determine the number of meters between each sampling location along the perimeter of the source. We continued around the perimeter of each source until we either reached our starting location, or we travelled a total of 50 meters. In order to ensure the predator communities sampled co-occur with *Culex* mosquito larvae, we sampled mosquito larvae used a similar approach with a random list of numbers between 1 and 3 to sample for mosquito larvae, using a 1.5 cup mosquito dipper to sample around the perimeter of each source.

We identified all aquatic surveyed insects to the lowest taxonomic level we were confident doing in the field. Owing to their diversity, we were not confident identifying many aquatic beetles past their family level in the field. We brought samples of morphospecies back to the lab for further identification to formulate a general idea of the predators present and to research possible functional differences in their predatory abilities, which is more within the scope of this study than a full taxonomic survey.

*Sex standardization of mosquito adults*

We sex-standardized for adult longevity and size (X) by measuring the distance of each individual mosquito’s trait from the average (X̄) of its respective sex’s longevity and size in control treatments for female (F) and male (M) insects:

*Sex-standardized trait value*= X_F_*_i_*- X̄ _F,control_ or X_M_*_i_*- X̄_M,control_
